# Management of a complex acetabular fracture following defibrillation for ventricular fibrillation cardiac arrest

**DOI:** 10.1136/bcr-2022-253421

**Published:** 2023-07-10

**Authors:** Samuel Watson, Theodora Bampouri, Ibraheim El-Daly, Kevin O’Gallagher

**Affiliations:** 1 School of Cardiovascular and Metabolic Medicine & Sciences, King's College London Faculty of Life Sciences and Medicine, London, UK; 2 Department of Cardiology, King's College Hospital NHS Foundation Trust, London, UK; 3 Department of Orthopaedic Surgery, King's College Hospital NHS Foundation Trust, London, UK

**Keywords:** Cardiovascular medicine, Interventional cardiology, Ischaemic heart disease, Drug therapy related to surgery, Orthopaedic and trauma surgery

## Abstract

In this case report, we describe the first case of a patient who sustained a complex acetabular fracture following defibrillation for ventricular fibrillation cardiac arrest in the context of acute myocardial infarction. The patient was unable to undergo definitive open reduction internal fixation surgery due to the need to continue dual antiplatelet therapy following coronary stenting of his occluded left anterior descending artery. Following multidisciplinary discussions, a staged approach was opted for, with percutaneous closed reduction screw fixation of the fracture performed while the patient was maintained on dual antiplatelet therapy. The patient was discharged with a plan to perform definitive surgical management when safe to discontinue dual antiplatelets. This is the first confirmed case of defibrillation causing an acetabular fracture. We discuss the various aspects that need to be considered when patients are being worked up for surgery while on dual antiplatelet therapy.

## Background

Primary percutaneous coronary intervention (PCI) with implantation of a drug-eluting stent is the gold-standard treatment for ST-elevation myocardial infarction (STEMI). Following this, patients are routinely treated with 12 months of dual antiplatelet therapy (DAPT), unless there is a bleeding contraindication. In patients who require non-cardiac surgery while on DAPT, several considerations need to be balanced to manage risks to patients and overall outcomes.

## Case presentation

A man in his 70s presented to our hospital via the primary acute myocardial infarction (MI) pathway having woken up at 04:00 with central chest pain radiating to both arms. His medical history included chronic kidney disease stage 3, hypothyroidism and osteoporosis with previous right inferior pubic ramus fracture 15 years previously. A 12-lead ECG demonstrated anterior ST elevation. He was loaded with 300 mg aspirin. During his transfer to hospital, he suffered a ventricular fibrillation cardiac arrest. Return of spontaneous circulation was achieved after a single cycle of cardiopulmonary resuscitation and a single direct current shock. Following this, he reported severe left leg pain.

## Differential diagnoses

The primary diagnosis was confirmed as anterior STEMI on the basis of persisting anterior ST elevation on 12-lead ECG and anterior wall hypokinesis on transthoracic echocardiography (TTE). Due to pain in multiple limbs (both arms and left leg) in the setting of chest pain, an acute aortic syndrome was considered as a differential; however, there were no echocardiographic features to support the diagnosis. On clinical examination, there was no radio-radial delay and on palpation of the femoral pulses, which were present and equal, there was extreme tenderness in the left groin. The patient’s left leg appeared shortened and externally rotated. Therefore, in addition to the anterior STEMI, there was a high clinical suspicion of hip fracture.

Given the clinical urgency of performing coronary angiography, the patient was given a second antiplatelet drug (prasugrel) and immediately taken to the cardiac catheter laboratory.

**Figure 1:**
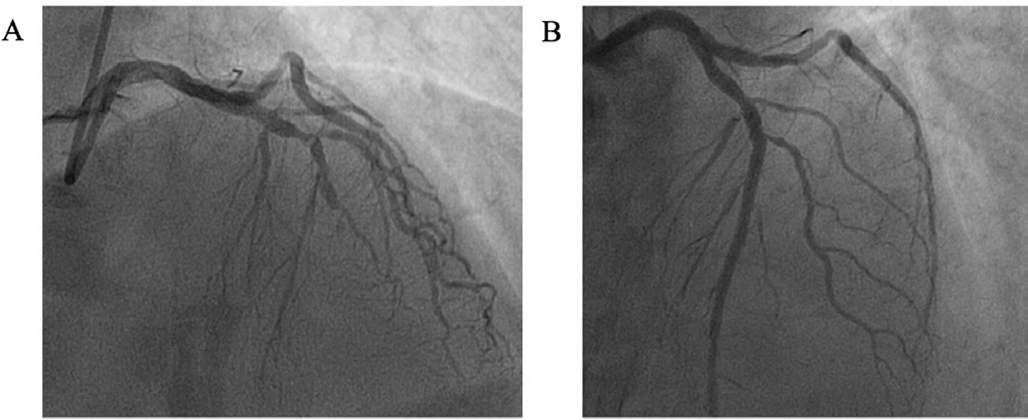
Coronary angiography. (A) Coronary angiogram demonstrating occlusion of the mid-left anterior descending coronary artery. (B) Coronary angiogram demonstrating TIMI 3 flow in the left anterior descending coronary artery after coronary stenting with a single drug-eluting stent to culprit lesion.

Coronary angiography demonstrated mid-left anterior descending (LAD) artery occlusion (figure 1A). Primary PCI to the culprit lesion with a single drug-eluting stent was performed, achieving an excellent angiographic result (figure 1B). Due to a large thrombus burden in the distal LAD, the patient was further treated with a glycoprotein IIB/IIIA infusion. There was no significant bystander coronary artery disease. The patient was started on DAPT. Formal TTE following PCI demonstrated preserved left ventricular ejection function (58%), with akinesis of the apex and septum.

**Figure 2:**
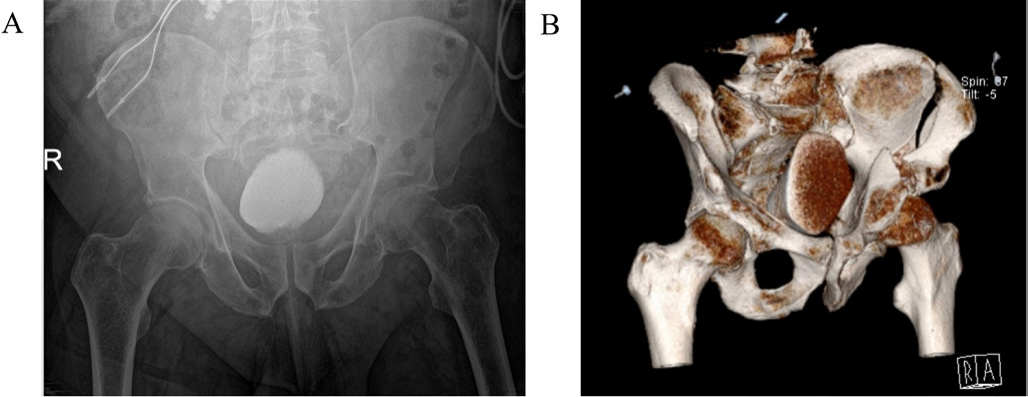
Radiological investigations for acetabular fracture. (A) Plain film radiograph demonstrating left-sided acetabular fracture, involving the anterior and superior columns. (B) Three-dimensional reconstruction of CT of the pelvis demonstrating a moderately displaced fracture of the left iliac bone, which extends from the left acetabulum, where there is a comminuted intra-articular fracture involving the acetabular root, anterior and posterior columns of the acetabulum and left proximal superior ramus.

Plain film radiograph of the left hip demonstrated a suspected acetabular fracture, likely involving both the anterior and posterior columns (figure 2A). The patient was referred to the orthopaedic team who requested a CT of the pelvis. Due to ongoing hip pain, the patient was given an ultrasound-guided fascia iliaca nerve block. CT of the pelvis demonstrated a moderately displaced left iliac wing fracture, extending from the iliac crest to the left acetabulum, where there was a comminuted intra-articular fracture involving the acetabular root, anterior and posterior columns of the acetabulum and left proximal superior pubic ramus (figure 2B). The patient was kept on bed rest and non-weight-bearing (NWB).

## Follow-up

Given the complexities associated with managing an acetabular fracture in the context of recent MI and DAPT, a multidisciplinary meeting was held between the cardiology and orthopaedic teams. From an orthopaedic perspective, the fracture would be best managed with open reduction internal fixation (ORIF) surgery within 2 weeks. However, a long operation (estimated 4–5 hours) in the context of recent MI and DAPT was considered high risk from both an anaesthetic and bleeding perspective. The cardiology team were happy for an operation to be considered 7 days following MI, which would have allowed surgical management within the proposed 2-week time frame. However, it was considered too risky to discontinue DAPT due to potential acute stent thrombosis, putting the patient at high surgical bleeding risk. An alternative conservative approach of 6–8 weeks of bed rest, and the associated potential complications of this, were also considered.

**Figure 3:**
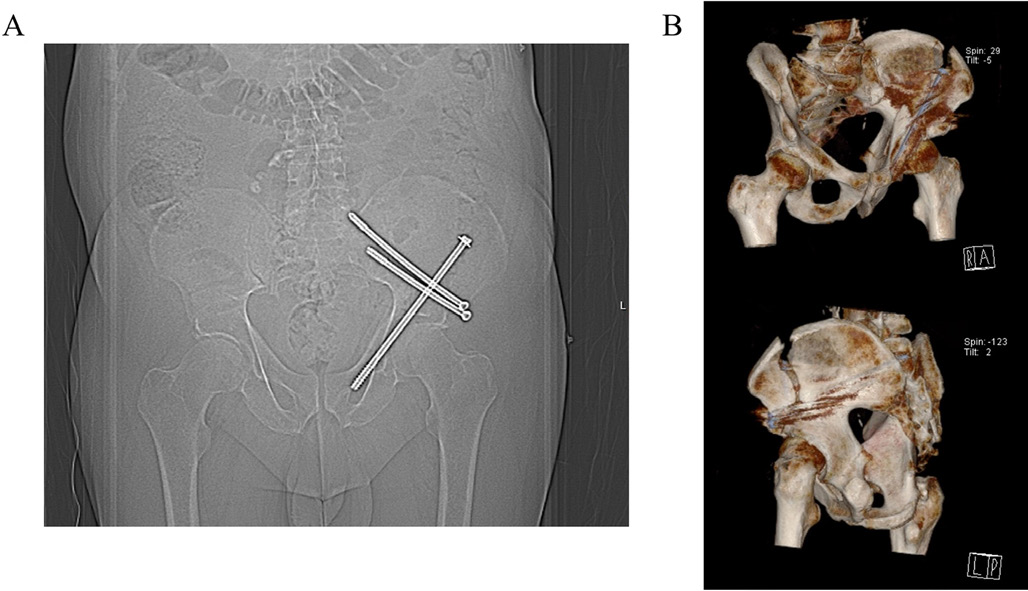
Closed reduction and screw fixation of left acetabular fracture. (A) Plain film radiograph demonstrating a left-sided long lag screw, with distal end lying within the superior pubic ramus. Further screws were placed through the supra-acetabular ilium. (B) Three-dimensional reconstruction of CT of the pelvis demonstrating a long lag screw and two supra-acetabular screws, as described above.

Given the risks associated with both ORIF and conservative management, a hybrid strategy of an initial CT-guided percutaneous closed reduction screw fixation of the fracture, providing pain relief and facilitating mobilisation, followed by a staged definitive total hip replacement in due course, were decided on. The procedure was successfully performed, with a long lag screw and two supra-acetabular screws stabilising the fracture (figure 3). Following the procedure, the patient had a significant reduction in his analgesia requirement. He was kept NWB on his left leg for 8 weeks, but was able to mobilise independently using a rollator frame. He was discharged 20 days after his MI and 6 days after his orthopaedic procedure.

The patient will be reviewed in orthopaedic clinic with a view to performing a total hip replacement when safe to discontinue DAPT. He will be routinely followed up by the cardiology team. Community cardiac rehabilitation and physiotherapy were arranged.

## Discussion

In this case report, we have described the management of a complex acetabular fracture following defibrillation due to acute MI-induced ventricular fibrillation cardiac arrest. We believe this to be the first confirmed case report of acetabular fracture secondary to defibrillation. Non-traumatic acetabular fractures are very rare, but can occur following forceful contraction of the pelvic muscles. A previous case report described an acetabular fracture in the context of both a tonic–clonic seizure and defibrillation following MI, but whether the fracture was secondary to either the seizure or cardioversion remained unknown.^1^ Other case reports have highlighted both subtrochanteric^2^ and trochanteric^3^ femoral fractures following electrical cardioversion. In all of these cases, patients had either osteoporosis or previous femoral fracture. In our case, the patient had both osteoporosis and a previous contralateral pelvic fracture, putting him at particularly high risk. This case highlights a small, but relevant, risk of fracture following defibrillation or cardioversion in patients with either osteoporosis or previous fracture, which should be investigated if patients reported pelvic/hip pain following a shock. In cases of ventricular fibrillation cardiac arrest, patients should receive immediate treatment including defibrillation as per Advanced Life Support guidelines^4^; however, the use of muscle relaxants may be considered in the context of elective cardioversion.^3^

Around 100 000 PCIs are performed in the UK each year.^5^ The European Society of Cardiology (ESC) recommends (IA recommendation) 12 months of DAPT, with a P2Y_12_ inhibitor on top of aspirin, for 12 months following acute MI with coronary stent implantation, to reduce the risk of both stent thrombosis and restenosis.^6^ The risk of stent thrombosis and restenosis is significantly lower with newer generation drug-eluting stents, such as was implanted in this case, but DAPT is still necessary. It has been estimated that between 5% and 25% of patients with coronary stents will require non-cardiac surgery within 5 years of stent implantation. For patients still on DAPT, several aspects must be considered. These include: (1) risk of stent thrombosis if DAPT is stopped, (2) complications associated with surgical delays while still on DAPT and (3) the bleeding risks associated with performing surgery on DAPT.^6^ In the acute phase following MI, the anaesthetic risk must also be considered. Optimal management strategies must consider and evaluate all of these variables, alongside patient preference.

In our case, the consensus decision was that given the acute timing of stent implantation, stopping DAPT would be very high risk for stent thrombosis. Given pelvic ORIF surgery is associated with high bleeding risk, the orthopaedic team were unable to proceed while the patient was on DAPT. Conservative management with 6–8 weeks of bed rest was considered, but was not deemed optimal as it would have left the patient in considerable pain and at risk of potentially life-threatening venous thromboembolism secondary to immobilisation. A hybrid staged approach was adopted, with the fracture stabilised percutaneously, which was performed with the patient on DAPT. There is a lack of high-quality evidence to identify a clear time frame for when patients can stop DAPT following coronary stenting with no or an acceptably low additional risk of undergoing surgery. In patients at high ischaemic risk due to a previous acute coronary syndrome presentation, the ESC recommends delaying surgery for 6 months following stent implantation if possible and the continuation of aspirin perioperatively if bleeding risk allows and to resume DAPT as soon as possible.^6^

**Learning points**

- Pelvic and femoral fractures are a rare, but recognised, complication of electrical cardioversion/defibrillation in patients with a background of osteoporosis or previous fracture.
- For patients who require non-cardiac surgery while on dual antiplatelet therapy (DAPT), (1) the risk of stent thrombosis if DAPT is stopped, (2) the risk of surgical delays associated with patients being on DAPT and (3) bleeding risks associated with surgery while on DAPT, must be considered.
- A multidisciplinary team approach is required when managing patients with multiple pathologies requiring intervention.
- Percutaneous closed reduction screw fixation of acetabular fractures can be performed while patients are on DAPT, and this can be used to bridge the required DAPT period prior to definitive surgical management.
